# Golden Snub-Nosed Monkeys: Potential Primate Paradigm in Post-Traumatic Stress Disorder Research

**DOI:** 10.3390/biology14020156

**Published:** 2025-02-03

**Authors:** Haitao Zhao, Yan Wang, Jiaxuan Li, Nianlong Li, Wenhui Zhou, Chengliang Wang, Baoguo Li

**Affiliations:** 1 Shaanxi Provincial Field Observation & Research Station for Golden Monkey, Giant Panda and Biodiversity, Shaanxi Institute of Zoology, Xi’an 710032, China; wy0218@xab.ac.cn (Y.W.); baoguoli@nwu.edu.cn (B.L.); 2 Shaanxi Key Laboratory for Animal Conservation, Northwest University, Xi’an 710069, China; nianlong@stumail.nwu.edu.cn (N.L.); zhouwenhuibio@163.com (W.Z.); 3 The School of Technology, Beijing Forestry University, Beijing 100091, China; 4 Graduate School of Management, University of California Davis, Davis, CA 95616, USA; 5 College of Life Science, Yanan University, Yanan 710032, China

**Keywords:** primate paradigm, male infanticide, trauma, post-traumatic stress disorder, social behavior

## Abstract

Post-traumatic stress disorder (PTSD) is a severe psychiatric disorder, affecting approximately 25–35% of individuals exposed to significant psychological trauma. Despite extensive research, the underlying biological mechanisms of PTSD remain poorly understood, and the effectiveness of trauma-focused psychotherapeutic interventions is limited. To date, animal studies investigating trauma responses have primarily relied on rodent models, with the potential contributions of non-human primates relatively neglected. In this study, matched-control observations were employed to examine maternal caregiving behaviors, prosocial interactions, and responses to alarm calls in free-ranging golden snub-nosed monkeys (*Rhinopithecus roxellana*) before and after an infanticide event. Results indicated a significant decrease in prosocial behaviors among some mothers, accompanied by significant increases in caregiving behaviors, heightened responsiveness to alarm calls, and indications of low mood. These findings provide evidence of trauma-related behavioral changes in golden snub-nosed monkeys, underscoring their utility as a model for studying PTSD given the similarity to trauma responses observed in humans. Overall, this study provides a foundation for further investigation into the evolutionary and biological aspects of PTSD, as well as the interactions between species-specific trauma responses. Future research should explore the use of non-human primate models to deepen our understanding of PTSD mechanisms.

## Simple Summary

Post-traumatic stress disorder (PTSD) is a severe psychiatric condition with high prevalence. Understanding how exposure to trauma manifests as PTSD—and whether individuals can recover naturally—has important implications for developing and improving exposure-based psychotherapeutic treatments. However, research on trauma recovery and natural self-regulation in non-human primates under naturalistic conditions remains limited, hindering the development of comparative models for natural psychotherapy and trauma recovery. To address this gap, the current study employed matched-control observations to examine maternal caregiving behaviors, prosocial interactions, and alarm responses in golden snub-nosed monkeys (*Rhinopithecus roxellana*). This study provides a novel perspective beyond traditional animal models in PTSD research, establishing this species as an ideal primate model for exploring the biological mechanisms underlying PTSD and advancing trauma-focused psychotherapeutic approaches.

## 1. Introduction

Post-traumatic stress disorder (PTSD) is a debilitating psychiatric condition characterized by a range of symptoms, including flashbacks, avoidance behaviors, negative emotional states, and hyperarousal, which persist for weeks to months following exposure to severe trauma [1,2]. The prevalence of PTSD is high, with rates reaching approximately 35% in groups subjected to severe psychological trauma [3]. Trauma often arises from extraordinary and distressing events, such as the sudden loss of a loved one, personal tragedy, or other life-altering incidents [4,5,6,7]. Currently, standard PTSD treatments primarily involve pharmacological and psychotherapeutic approaches. While pharmacotherapies can alleviate symptoms related to depression, arousal, and anxiety [8], trauma-focused psychotherapy remains the most effective intervention for managing and mitigating PTSD symptoms [1]. Understanding how exposure to trauma manifests as PTSD—and whether individuals can recover naturally—has important implications for developing and improving exposure-based psychotherapeutic treatments [9].

Research on PTSD and trauma recovery has been extensively conducted in controlled animal models. These studies have confirmed that threat-related behaviors and their underlying neural circuitry are highly conserved across species, from rodents to humans [1,10,11]. However, investigations into trauma recovery and natural self-regulation of PTSD in mammalian models, particularly under naturalistic conditions, remain limited. The absence of such studies, especially in non-human primates, hinders the development of comparative models for natural psychotherapy and trauma recovery. Non-human primates, with their advanced cognitive abilities, complex social structures, and shared physiological traits with humans, represent an ideal model for exploring PTSD mechanisms [12]. Investigating whether non-human primates experience PTSD and how they self-regulate trauma responses under natural conditions offers a unique opportunity to uncover biologically grounded strategies for PTSD recovery. Such research holds promise for informing and enhancing psychotherapeutic approaches to human PTSD.

Golden snub-nosed monkeys (*Rhinopithecus roxellana*), an Asian colobine species endemic to China, are renowned for their highly social nature, with a complex, multi-level social structure comprising multiple one-male units (OMUs) and several all-male units (AMUs) [13,14]. Within this framework, male takeovers of OMUs are frequent and often associated with infanticide [15,16]. During these events, mothers display immediate counterstrategies to protect their offspring, such as maintaining proximity to the ousted male or seeking refuge with him following an attack. Despite these efforts, unweaned infants can still be killed, with affected mothers exhibiting profoundly despondent behaviors, such as holding their deceased infants and isolating themselves from their families for extended periods [17]. These maternal responses suggest a heightened capacity for cognitive empathy in *R. roxellana*, especially compared to other Old World monkeys. This cognitive ability, coupled with the complex social structure of this species, reveals parallels with human emotional responses to trauma, as documented in previous studies [4,18]. These findings support the hypothesis that *R. roxellana* may experience PTSD-like symptoms. Furthermore, the self-regulation strategies employed by this species to cope with trauma may offer valuable insights into developing therapeutic approaches for treating PTSD in humans.

## 2. Materials and Methods

### 2.1. Study Site and Species

This study focused on a provisioned troop of golden snub-nosed monkeys inhabiting the Dapingyu region of Guanyinshan National Nature Reserve on the southern slopes of the Qinling Mountains, Shaanxi Province, China (107.98–107.99°E, 33.66–33.71°N). From 8:30 am to 5:30 pm on each day of observation, the troop was herded to a designated 20 m × 25 m area in Erdaohe (1530 m a.s.l.) within Yanjiagou Valley. Provisioning occurred three times daily at 9:00 am, 12:00 pm, and 3:00 pm, with apples, radishes, and corn distributed randomly to minimize interference with natural behaviors. Outside provisioning times, the monkeys moved freely within their home range. Although provisioning likely influenced their daily activities and social interactions initially, the monkeys soon became well-habituated to human presence, and largely ignored the researchers, enabling observations at distances of 5–20 m. To minimize the impact of artificial herding on their social behaviors, observation periods were restricted to between 9:00 am and 5:00 pm, coinciding with the peak activity of the troop during this time frame [19]. Individual adult monkeys were identified based on distinct physical characteristics, such as coloration, facial features, body size, and scars [20]. The size of the focal troop increased from 42 individuals in September 2014, comprising four one-male, multi-female units (OMUs) and three individuals in one all-male unit (AMU), to 87 individuals in December 2023, comprising seven OMUs and 19 individuals in two AMUs. Golden snub-nosed monkeys exhibit a complex, multi-level social structure similar to that observed in human societies. Males from AMUs typically range along the periphery of the troop and periodically attempt to invade an OMU, seeking opportunities to replace the resident leader male [21].

### 2.2. Data Collection

Data were collected daily from September 2014 to December 2023 between 9:00 am and 5:00 pm at distances of 5–20 m [12,22,23]. Adult individuals within each OMU were systematically selected as focal subjects for daily observations. To assess PTSD-like symptoms in golden snub-nosed monkeys, behavioral criteria were adapted from the three primary symptom domains of PTSD in humans [24]: (1) responses to alarm calls, representing hyperarousal symptoms such as heightened vigilance, irritability, and being on guard without actual danger; (2) maternal behaviors, reflecting re-experiencing of trauma, often triggered by specific events, such as male takeovers or infanticide attempts; and (3) prosocial behaviors, characterized by withdrawal from social interactions and isolation from other members of the same OMU. Symptoms of PTSD in golden snub-nosed monkeys were considered analogous to human PTSD, appearing within one month of a traumatic event and resulting in heightened stress and anxiety even in the absence of danger.

Matched-control observations were employed to systematically assess maternal prosocial behaviors, caregiving behaviors, and alarm-call responses. Male takeover and infanticide events were recorded using all-occurrence sampling, while focal animal sampling was utilized to continuously document maternal social behaviors and alarm-call responses following male takeovers. After male replacement, maternal caregiving behaviors (e.g., carrying, holding, and grooming) and prosocial interactions (e.g., embracing, allomaternal care, and grooming) were analyzed using instantaneous scan sampling at 10 min intervals. In cases where infanticide occurred following male takeovers, maternal responses to alarm calls, caregiving behaviors, and prosocial interactions were also observed for 30 days using the same methodology to evaluate post-infanticide behavioral patterns. The male replacement was confirmed when the ousted male either permanently left the original unit or remained on its periphery without engaging, allowing copulations between the new male and females to proceed without disruption [18]. Previous research indicates that male replacements predominantly occur between December and March, with infanticide linked to increased mating opportunities for the incoming male. Early infanticide events often take place approximately 30 days after a male replacement [25]. Therefore, to account for the adjustment period between mothers and the ousted male, behavioral data were collected for 30 days before and 30 days after infanticide events, serving as comparative observation and analysis periods. Here, infanticide events were defined as either “successful”, where the infant was killed, or “attempted”, where the infant survived the attack.

For paternity analysis, 5–6 tail-root hair samples were non-invasively collected from all infants born during the study period, their mothers, and all adult males within the focal troop [12]. Mother–infant dyads were identified based on direct observations of maternal caregiving behaviors, such as feeding and carrying. Each individual was sampled at least twice to ensure the reliability of genetic data. DNA was extracted from hair follicles using an Ezup Column Animal Genomic DNA Purification Kit (B518251, Sangon Biotech, Shanghai, China). Paternity testing was performed using 18 highly polymorphic microsatellite loci (D3s1766, D6s501, D6s1040, D7s820, D7s1804, D7s2204, D8s1049, D9s252, D10s676, D10s1432, D10s2483, D12s375, D14s306, D18s1371, D19s248, D19s582, D19s1034, and D21s2054). Microsatellite loci analysis, polymerase chain reaction (PCR) amplification, and genotyping were performed following previously described protocols [26]. Simple sequence repeat (SSR) data were analyzed using GeneMapper 5 software, and paternity was assessed using PARENTAGE [27], enabling confirmation of kinship relationships.

### 2.3. Data Analysis

All data were derived from multi-year focal animal sampling and instantaneous scan sampling observations of the social behaviors of mothers with infants. Data analysis for each sampling session was classified into three stages before and after the infanticide events: Period 1 (days 1–10 before infanticide), Period 2 (days 11–20 before infanticide), Period 3 (days 21–30 before infanticide), Matched period 1 (days 1–10 after infanticide), Matched period 2 (days 11–20 after infanticide), and Matched period 3 (days 21–30 after infanticide). Behavioral data for each mother involved in an infanticide event were systematically analyzed and classified into two groups: successful infanticide group (infant died following an attack by the new male) and attempted infanticide group (infant survived the initial attack by the new male). Data management and analysis were performed using Microsoft Excel and SPSS v16.0 (SPSS Inc., Chicago, IL, USA).

To examine behavioral differences in mothers before and after infanticide events, as well as between the successful infanticide group and attempted infanticide group, the Wilcoxon matched-pairs signed-rank test was used. Behavioral changes in mothers across the three matched periods (1–3) after infanticide were assessed using the Friedman test. Statistical significance was determined at a threshold of *p* < 0.05.

## 3. Results

Over a 10-year observation period, 21 infanticide cases were recorded following male replacements, including 5 resulting in infant deaths. Maternal behaviors were systematically extracted from the 30-day periods before and after each infanticide case, focusing on prosocial behaviors, caregiving behaviors, and alarm-call responses across multi-year field observations. The frequencies of these behaviors were statistically analyzed, as shown in Table 1. A total of 1288 days of observations were collected from 21 mother–infant pairs, averaging 36 effective observation days per case (6 days per period).

Behavioral differences in mothers with infants were assessed across three periods before and three matched periods after infanticide events using the Wilcoxon matched-pairs signed-rank test. Statistical analyses revealed significant differences in maternal behavior overall. Specifically, caregiving behaviors and responses to alarm calls significantly increased, whereas prosocial behaviors significantly decreased (prosocial behavior: *N* = 378, *t* = 15.30, *p* < 0.01; caregiving behavior: *N* = 288, *t* = −18.37, *p* < 0.01; alarm response: *N* = 378, *t* = −14.94, *p* < 0.01). In 16 attempted infanticide cases, maternal caregiving and alarm-call responses also increased, with a concurrent decrease in prosocial behaviors (prosocial behavior: *N* = 288, *t* = 18.67, *p* < 0.01; caregiving behavior: *N* = 288, *t* = −18.37, *p* < 0.01; alarm response: *N* = 288, *t* = −17.83, *p* < 0.01). In contrast, among the five successful infanticide cases, no significant differences in maternal prosocial behaviors or alarm-call responses were observed (prosocial behavior: *N* = 90, *t* = 3.22, *p* = 0.06 > 0.05; alarm response: *N* = 90, *t* = −0.70, *p* = 0.48 > 0.05). Notably, in some cases, mothers abandoned their deceased infants within a few days of the infanticide event, which impacted caregiving behaviors and led to their cessation.

The Friedman test was applied to assess behavioral changes among mothers in the three matched periods (1–3) following infanticide events. In the 16 cases of attempted infanticide, no significant differences were observed in the frequencies of prosocial behaviors, caregiving behaviors, or alarm-call responses over time (prosocial behavior: *N* = 96, *df* = 2, *p* = 0.89 > 0.05; caregiving behavior: *N* = 96, *df* = 2, *p* = 0.97 > 0.05; alarm response: *N* = 96, *df* = 2, *p* = 0.89 > 0.05). In contrast, the five cases of successful infanticide revealed significant variability in maternal behavior across the post-infanticide periods. Prosocial behaviors and alarm-call responses both exhibited notable fluctuations, suggesting distinct behavioral adaptations or heightened stress responses following these traumatic events (prosocial behavior: *N* = 30, *df* = 2, *p* = 0.002 < 0.05; alarm response: *N* = 30, *df* = 2, *p* = 0.03 < 0.05).

## 4. Discussion

This study investigated whether traumatic events, such as infanticide, induce PTSD-like symptoms in golden snub-nosed monkey mothers and explored their capacity for self-regulation following such trauma. The findings offer valuable insights into the biological mechanisms underlying natural recovery processes, providing a foundation for understanding therapeutic targets for intervention in human PTSD. Historically, traumatic research has largely been restricted to rodent models [1,11,28]. While these models have elucidated conserved neural mechanisms, their limited physiological and social parallels with humans constrain their applicability to understanding PTSD. In contrast, non-human primates exhibit complex social structures, more established brain and advanced cognitive abilities, and trauma-related biological responses similar to humans, making them ideal candidates for comparative PTSD research. Investigating the behavioral and self-regulatory mechanisms of trauma in non-human primates provides critical insights into the associations between trauma, social structure, social recognition, and external environmental pressures.

During infanticide events, mothers exhibited various counterstrategies, such as avoiding new males or withdrawing from other members of their original OMU. These behaviors suggest heightened fear responses and negative emotional states, potentially contributing to the onset of PTSD-like symptoms [18]. Data from this 10-year comparative study revealed that trauma following infanticide events significantly impacted maternal behavior in golden snub-nosed monkeys. Between three periods before and three matched periods after infanticide, maternal prosocial interactions with other females within the same OMUs significantly decreased, while caregiving behaviors and alarm-call responses increased. In the 16 cases where infants survived infanticide attempts, mothers displayed persistent behavioral changes. Prosocial behaviors remained suppressed, and caregiving and alarm-call responses were consistently elevated, indicating sustained anxiety and hyper-vigilance. These sustained responses likely stem from an ongoing fear of future attacks and the prolonged stress associated with perceived threats, even in the absence of immediate danger. Such patterns are strikingly similar to chronic PTSD in humans, where traumatic stress often manifests as hyper-vigilance, increased protective behaviors, and impaired social engagement. The adage “once bitten, twice shy” aptly characterizes this prolonged state of caution. In contrast, in the five mother–infant pairs where infants were killed, maternal behavior exhibited no significant differences between the pre- and post-infanticide observation periods. This suggests that while mothers experienced initial traumatic responses, many individuals recovered naturally within 30 days.

Notably, behavioral recovery patterns differed between mothers in the successful and attempted infanticide groups during the three matched periods (1–3) following infanticide events. Results revealed that most mothers in the successful infanticide group exhibited clear signs of behavioral recovery by the end of the 30-day observation period. In contrast, mothers in the attempted infanticide group showed no comparable recovery, likely due to repeated threats and ongoing exposure to stressful events. Such repeated trauma appears to intensify stress responses, similar to the compounding effects of chronic trauma observed in humans [29]. These findings suggest that short-term trauma may be more conducive to natural recovery, as it allows individuals to adapt and return to baseline behavior more effectively, reflecting the saying “better a little loss than a long sorrow”. In cases of successful infanticide, rapid adaptive responses may mitigate prolonged psychological distress. This resilience may be attributed to the social structure and cognitive ability of the species. In golden snub-nosed monkeys, female offspring often remain in their natal OMU, forming strong social bonds with their mothers and other related females [30]. Such alliances likely provide social buffering that mitigates the effects of trauma. Furthermore, unique behavioral adaptations in this species, such as dual caregiving of infants—one of which is not biologically related to the dominant male—may have evolved as a strategy to reduce infanticide risk [12]. This cognitive capacity and social complexity enhance the ability to cope with trauma and demonstrate parallels with human social resilience mechanisms.

While multiple factors influence PTSD susceptibility, including personal history, age, sex, psychological state, and trauma type, there is considerable overlap in the biological mechanisms of trauma responses across humans and non-human primates [6]. This study established the potential utility of non-human primates as a valuable model for investigating PTSD in humans, highlighting behavioral similarities in responses to traumatic events and their relevance to advancing the treatment of human PTSD. However, ethical considerations in studying trauma in non-human primates necessitate caution. Future research should prioritize the identification of specific biological mechanisms that can be targeted through evidence-based cognitive behavioral therapies and the exploration of effective strategies for trauma regulation, for example, we will continue to focus on the comparison between mothers in the successful and attempted infanticide groups relying on whether memories of their previous loss are recalled when these mothers have children in the future. Direct investigations into the underlying processes of PTSD, such as hormonal fluctuations, brain activity, and genetic markers in non-human primates, are not recommended due to ethical concerns. While primate models cannot fully capture the complexity of human experience, they offer critical insights into trauma responses where human studies fall short. Integrating findings from primate research with human studies offers a complementary approach that bridges the gaps in understanding PTSD, enabling the development of more effective and ethically sound treatment strategies.

## 5. Conclusions

This study provides evidence of trauma-related behavioral changes in golden snub-nosed monkeys, underscoring their utility as a model for studying PTSD given the similarity to trauma responses observed in humans.

## Figures and Tables

**Table 1:** Frequency of prosocial behaviors, caregiving behaviors, and alarm-call responses in mothers across three periods before and three matched periods after infanticide events.

Infanticide Event	Behavioral Patterns of Mothers	Sampling Session					
		Before Infanticide			After Infanticide		
		Period 1 (Days 1–10)	Period 2 (Days 11–20)	Period 3 (Days 21–30)	Matched Period 1 (Days 1–10)	Matched Period 2 (Days 11–20)	Matched Period 3 (Days 21–30)
Attempted infanticide group	Caregiving behaviors (mean ± SE)	6.02 ± 3.31	6.19 ± 2.83	6.40 ± 2.84	11.61 ± 4.77	11.92 ± 4.92	12.35 ± 4.00
	Alarm-call responses (mean ± SE)	0.44 ± 0.18	0.45 ± 0.16	0.44 ± 0.17	0.68 ± 0.17	0.69 ± 0.17	0.69 ± 0.16
	Prosocial behaviors (mean ± SE)	14.88 ± 5.47	15.23 ± 5.78	15.52 ± 5.60	8.55 ± 3.37	8.79 ± 3.24	8.88 ± 3.10
Successful infanticide group	Caregiving behaviors (mean ± SE)	-	-	-	-	-	-
	Alarm-call responses (mean ± SE)	0.46 ± 0.14	0.46 ± 0.16	0.46 ± 0.15	0.53 ± 0.18	0.48 ± 0.12	0.42 ± 0.17
	Prosocial behaviors (mean ± SE)	14.90 ± 5.74	14.53 ± 5.49	13.40 ± 5.63	11.73 ± 3.69	10.97 ± 5.26	15.47 ± 5.59

## Data Availability

The original data records and other requirements are available by contacting the corresponding or first authors.

## References

[1] Ressler K.J. (2020). Translating across circuits and genetics toward progress in fear- and anxiety-related disorders. Am. J. Psychiatry.

[2] Harvey A.R. (2023). Injury, illness, and emotion: A review of the motivational continuumfrom trauma through recovery from an ecological perspective. Brain Behav. Immun..

[3] McLaughlin K.A., Koenen K.C., Friedman M.J., Ruscio A.M., Karam E.G., Shahly V., Stein D.J., Hill E.D., Petukhova M., Alonso J. (2015). Subthreshold posttraumatic stress disorder in the world health organization world mental health surveys. Biol. Psychiatry.

[4] McGrath J.J., McLaughlin K., Saha S., Aguilar-Gaxiola S., Al-Hamzawi A., Alonso J., Bruffaerts R., de Girolamo G., de Jonge P., Esan O. (2017). The association between childhood adversities and subsequent first onset of psychotic experiences: A cross-national analysis of 23,998 respondents from 17 countries. Psychol. Med..

[5] Ferris L.J., Jetten J., Hornsey M.J., Bastian B. (2019). Feeling hurt: Revisiting the relationship between social and physical pain. Rev. Gen. Psychol..

[6] Wright L.A., Roberts N.P., Lewis C., Simon N., Hyland P., Ho G.W.K., McElroy E., Bisson J.I. (2021). High prevalence of somatisation in ICD-11 complex PTSD: A cross sectional cohort study. J. Psychosom. Res..

[7] Morin E.L., Siebert E.R., Howell B.R., Higgins M., Jovanovic T., Kazama A.M., Sanchez M.M. (2024). Effects of early maternal care on anxiety and threat learning in adolescent nonhuman primates. Dev. Cogn. Neurosci..

[8] Abdallah C.G., Averill L.A., Akiki T.J., Raza M., Averill C., Gomaa H., Adikey A., Krystal J.H. (2019). The neurobiology and pharmacotherapy of posttraumatic stress disorder. Annu. Rev. Pharmacol. Toxicol..

[9] Lewis C., Roberts N.P., Andrew M., Starling E., Bisson J.I. (2020). Psychological therapies for post-traumatic stress disorder in adults: Systematic review and meta-analysis. Eur. J. Psychotraumatol..

[10] Rosen A.M., Spellman T., Gordon J.A. (2015). Electrophysiological endophenotypes in rodent models of schizophrenia and psychosis. Biol. Psychiatry.

[11] Fenster R.J., Lebois L.A.M., Ressler K.J., Suh J. (2018). Brain circuit dysfunction in post-traumatic stress disorder: From mouse to man. Nat. Rev. Neurosci..

[12] Zhao H.T., Li J.X., Wang Y., Li N.L., Pan R., Li B.G. (2024). A Unique Case of Adoption in Golden Snub-Nosed Monkeys. Animals.

[13] Qi X.G., Garber P.A., Ji W.H., Huang Z.P., Huang K., Li B.G. (2014). Satellite telemetry and social modeling offer new insights into the origin of primate multilevel societies. Nat. Commun..

[14] Qi X.G., Wu J.W., Zhao L., Wang L., Guan X.M., Garber P.A., Opie C., Yuan Y., Diao R.J., Li G. (2023). Adaptations to a cold climate promoted social evolution in Asian colobine primates. Science.

[15] Yao H., Yu H.L., Yang B.H., Yang W.J., Xu H.Q., Grueter C.C., Li M., Xiang Z.F. (2016). Male Infanticide in the Golden Snub-Nosed Monkey (*Rhinopithecus roxellana*), a Seasonally Breeding Primate. Int. J. Primatol..

[16] Li W., Dong S.X., Niu F., Li N.L., Su Z.Y., Wang C.L., Huang K., Zhao H.T., Pan R.L., Zhang P. (2024). Infanticide in golden snub-nosed monkeys with multilevel society. Curr. Zool..

[17] Guo D., Qi X.G., Tian J.S., Hu Y.L., Si K.C., Gao C.L., Li B.G. (2016). Carrying of dead infants by golden snub-nosed monkeys (*Rhinopithecus roxellanna*) in the Qinling Mountains. Acta Theriol. Sin..

[18] Xiang Z.F., Yu Y., Yao H., Hu Q.L., Yang W.J., Li M. (2022). Female countertactics to male feticide and infanticide in a multilevel primate society. Behav. Ecol..

[19] Lv J.Q., Li B.G. (2006). Diurnal activity budgets of the Sichuan snub-nosed monkey (*Rhinopithecus roxellanna*) in the Qinling Mountains of China. Acta Theriol. Sin..

[20] Qi X.G., Li B.G., Ji W.H. (2008). Reproductive parameters of wild female *Rhinopithecus roxellanna*. Am. J. Primatol..

[21] Xiang Z., Yang B., Yu Y., Yao H., Grueter C.C., Garber P.A., Li M. (2014). Males collectively defend their one-male units against bachelor males in a multi level primate society. Am. J. Primatol..

[22] Zhao H.T., Wang X.W., Li J.X., Zhang J., Wang C.L., Qi X.G., Guo S.T., Wang R.T., Shi K., Wang X.Y. (2016). Postconflict behavior among *Rhinopithecus roxellana* leader males in the Qinling Mountain, China. Curr. Zool..

[23] Zhao H.T., Li J.X., Wang Y., Li N.L., Wang X.W., Wang C.L., Ren Y., Jia T., Pan R.L., Li B.G. (2021). Sexual interference and allomaternal behavior as predictors of rank recognition in female golden snub-nosed monkeys. Curr. Zool..

[24] Ressler K.J., Berretta S., Bolshakov V.Y., Rosso I.M., Meloni E.G., Rauch S.L., Carlezon W.A. (2022). Post-traumatic stress disorder: Clinical and translational neuroscience from cells to circuits. Nat. Rev. Neurol..

[25] Fang G., Chen J., Pan R., Qi X., Li B. (2018). Female choice impacts residential male takeover in golden snub-nosed monkeys (*Rhinopithecus roxellana*). Zool Res..

[26] Zhang P., Song X.Y., Dunn D.W., Li B.G. (2016). Diversity at two genetic loci associated with the major histocompatibility complex in the golden snub-nosed monkey. Biochem. Syst. Ecol..

[27] Huang K., Mi R., Dunn D.W., Wang T.C., Li B.G. (2018). Performing parentage analysis in the presence of inbreeding and null alleles. Genetics.

[28] Tryon V.L., Garman H.D., Loewy R.L., Niendam T.A. (2021). Links Between Human and Animal Models of Trauma and Psychosis: A Narrative Review. Biol. Psychiatry Cogn. Neurosci. Neuroimaging.

[29] Stevens L., Bregulla M., Scheele D. (2024). Out of touch? How trauma shapes the experience of social touch—Neural and endocrine pathways. Neurosci. Biobehav. Rev..

[30] Li Y.L., Wang L., Wu J.W., Ye X.P., Garber P.A., Yan Y., Liu J.H., Li B.G., Qi X.G. (2020). Bachelor groups in primates multilevel society facilitate gene flow across fragmented habitats. Curr. Zool..

